# The Transformation of Open Fractures into Closed Fractures

**Published:** 1917-11

**Authors:** 


***The Transformation of Open Fractures into Closed Fractures.*** Trans. of abstract in the *Archives de Médecine et de Pharmacie*, June 1917, p. 844. From a Communication to the Society of Surgery of Paris, at its meeting on June 11, 1917, published in the *Bulletin et Mémoires de la Société*, t. XLIII, N° 26, p. 1546.

M. Lagoutte has presented to the society the results of his experiences with a series ot open fractures of the thigh treated by immediate suture and thus transformed into closed fractures.

M. Lagoutte attempted suture in 7 cases. He was successful in 4, cleansing the wound simply without antiseptics. The wounds varied in age from 4 to 49 hours. The operation failed in 3 cases in which the wounds varied in age from 22 to 42 hours. The failure in these cases was due to the fact that infection had already set in at the time of the treatment; no injury to the patient, however, resulted from the operation. Of the 4 successful cases, 3 consolidated in from 25 to 42 days; the 4th was still in process of consolidation at the time the paper was presented. In the 3 fistulous cases the consolidation was somewhat slower, in from 35 to 45 days.

The technique is simple. After radioscopic examination to determine the presence of projectiles and of bone fragments, the wound is cleansed by the proper incisions, all the injured tissues along the tract are removed, and the bony lesions thoroughly cleaned while the seat of the injury *is kept clearly in view*. All loose splinters are removed; adherent splinters are not disturbed, except those which are not firmly attached, the vitality of which appears doubtful. The cleansing should be completed with the curette. The marrow in particular in curetted carefully at the extremities of the fractured bone, and along the internal face of the splinters left in position. All of the periostium, however, should be left undisturbed. All clots are then removed, and the wound dried by means of compresses held by forceps.

The first principle to be observed in the treatment of the wound after the operation is : never to admit in the region of the fracture any foreign substance, whether a drain, or folds of gauze. In order to provide drainage for the blood or serous discharge following the operation, it suffices merely to avoid an hermetically close suture. If at the end of 48 hours there is no temperature and no local reaction, the stitches left loose may be tightened so as to close the opening entirely. In all cases immobilization was accomplished by the apparatus styled " American ".

As for fistulous cases, a simple secondary operation suffices : it may consist of the removal of splinters or projectiles which were allowed to remain in place. Whatever the method of the operation the profuse and interminable suppuration is likely to disappear.

